# The 2SP Site Mutation in the Bovine Natural Resistance-Associated Macrophage 1 Promoter Exhibits Antituberculosis Potential

**DOI:** 10.3390/ijms26094229

**Published:** 2025-04-29

**Authors:** Yongke Wei, Mengke Yuan, Yong Zhang, Yuanpeng Gao

**Affiliations:** 1 College of Veterinary Medicine, Northwest A&F University, Yangling 712100, China; weitom2012@163.com (Y.W.); m15291999052@163.com (M.Y.); 2 Key Laboratory of Animal Biotechnology, Northwest A&F University, Yangling 712100, China

**Keywords:** base editing, NRAMP1, promoter analysis, tuberculosis

## Abstract

Gene-edited cattle overexpressing natural resistance-associated macrophage 1 (NRAMP1) have demonstrated enhanced resistance to tuberculosis (TB). However, introducing synthetic sequences and selection markers may pose potential risks. The endogenous editing of target gene promoters could effectively mitigate these risks. To date, no available mutation sites in the bovine *NRAMP1* promoter have been identified to enhance host resistance to TB. In this study, we identified a unique mutation editing site, designated as 2SP, within the bovine *NRAMP1* promoter, using bioinformatics analysis and dual luciferase assays. The mutation at the 2SP site specifically increased *NRAMP1* promoter activity by 2.3-fold after *Mycobacterium tuberculosis* H37Ra infection, without modifying promoter activity in non-infected groups. By using base editing techniques, an endogenously edited THP-1 cell line with a mutation at the homologous region of the 2SP site was generated, without introducing screening markers. In H37Ra infection experiments, the edited THP-1 cells specifically upregulated NRAMP1 expression and significantly inhibited H37Ra proliferation, while maintaining baseline NRAMP1 expression levels in the absence of infection. In this research, we identified a novel mutation site and provided a fundamental reference for the development of gene-edited cattle with enhanced resistance to TB.

## 1. Introduction

Tuberculosis (TB), mainly caused by *Mycobacterium tuberculosis* (Mtb), remains one of the most prevalent infectious diseases worldwide. Approximately 25% of the global human population exhibits immunological evidence of prior Mtb infection, and approximately 10% of infected individuals develop clinical disease during their lifetime [1].

The fact that only a small number of individuals infected with Mtb progress to TB indicates that genetic factors play a crucial role in resistance to TB, as affirmed by numerous studies [2,3,4,5,6,7,8,9]. *Mycobacterium bovis* (*M. bovis*) is the most prevalent pathogen among mycobacteria [10]. As a zoonotic disease, bovine TB not only has serious implications for animal production but also poses substantial risks to public health [11].

The natural resistance-associated macrophage 1 (*NRAMP1*) gene, also known as the solute carrier family 11 member 1 gene, is one of the most extensively investigated genes related to TB resistance [12]. *NRAMP1* was initially identified through positional cloning as a candidate gene for resistance against *M. bovis*, *Leishmania donovani*, and *lethal Salmonella typhimurium* [13]. These intracellular pathogens share survival strategies and replicate within mildly acidic macrophage vacuoles [14]. *NRAMP1* is specifically expressed in the membrane of late endosomes/lysosomes of professional phagocytes, functioning as a divalent cation transporter [15,16,17].

Although the related mechanisms remain to be further explored, genetic variation in *NRAMP1* is associated with susceptibility/resistance to TB [18,19,20,21,22], and it is considered to play a crucial role in resisting TB development [23]. The structure of *NRAMP1* and its function in preventing intracellular pathogen infections are both ancient and conserved [24], as evidenced by their analogous roles in natural resistance to intracellular pathogens, ranging from amoeba *D. discoideum* [25] to mouse macrophages [13].

TB resistance has been correlated with *NRAMP1* gene polymorphisms. The minor allele frequency (MAF) of genetic polymorphisms occurring in the exon of *NRAMP1* is relatively low, and these polymorphisms are frequently identified as risk factors [18,26,27,28,29]. Conversely, the MAF of polymorphisms located in regulatory regions of *NRAMP1* is comparatively higher and may confer positive effects [18,30,31,32]. Hence, gene editing targeting the regulatory regions to enhance a host’s resistance to TB may represent a more rational strategy.

Our previous research successfully produced gene-edited cattle with enhanced TB resistance by overexpressing NRAMP1 [33,34]. However, the introduction of synthetic sequences and marker genes poses potential risks. In this study, we report a novel mutation site available for endogenous base editing, designated as 2SP, and offer preliminary evidence of its distinctive effect on the regulation of bovine *NRAMP1* expression. As an unconventional variant recognition site for the *NRAMP1* transcription factors (TFs) SP1/SP3, the 2SP site exhibits a weakened affinity for SP1/SP3. We discovered that SP1/SP3 are TFs that activate *NRAMP1* expression. Nevertheless, increasing the affinity for SP1/SP3 at the 2SP site results in a significant downregulation of NRAMP1 expression. Interestingly, mutating the 2SP site to eliminate its affinity significantly enhances the promoter activity after H37Ra infection while leaving the promoter activity unchanged in the non-infected group.

Owing to the deficiency of bovine macrophage cell lines and based on the similarity in promoter sequences between bovine and humans, we established a 2SP site homologous region-edited THP-1 cell line for testing. The edited THP-1 cell line exhibited increased NRAMP1 protein expression after H37Ra infection and more effective inhibition of H37Ra proliferation.

## 2. Results

### 2.1. Detection of the Core Promoter of Bovine NRAMP1 and Comparative Analysis Between Mouse and Cattle

By integrating and analyzing RNA sequencing data from multiple independent laboratories deposited in the NCBI Gene Expression Omnibus profiles (https://www.ncbi.nlm.nih.gov/geoprofiles/, accessed on 19 June 2022), we observed that Mtb infection induced a substantial increase in Nramp1 expression in mice, whereas the induction in cattle was comparatively limited (Supplementary Figure S1A–D). In this study, the mouse macrophage cell line RAW264.7 and Mtb strain H37Ra were used to verify the results and for further experiments. In the subsequent experiments, we utilized a multiplicity of infection (MOI) of 10 to ensure the comparability and consistency of our experimental results with the analyzed datasets. This MOI was chosen to match the experimental conditions predominantly reported in the analyzed transcriptomic datasets. In RAW264.7 cells, *Nramp1* expression was significantly upregulated at 4 h post-infection (hpi), peaked at 12 hpi, and returned to baseline levels by 36 hpi (Supplementary Figure S1E).

**Figure 1:**
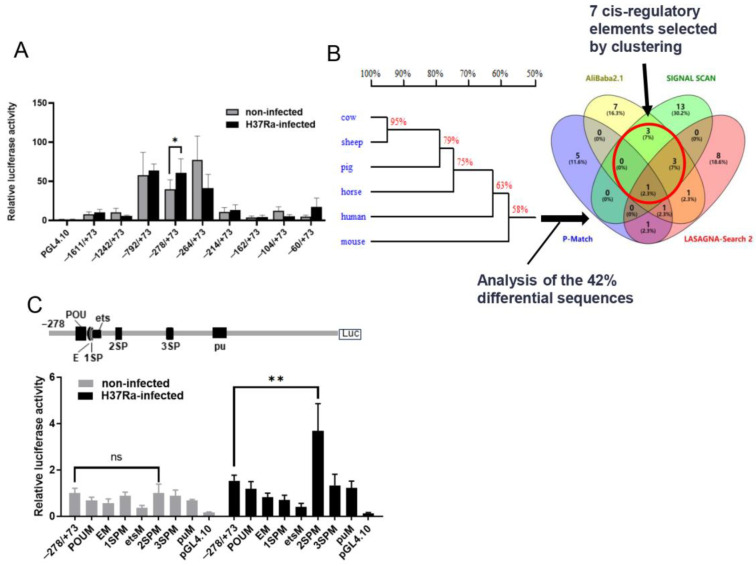
Analysis of the regulatory region of the *NRAMP1* promoter in cattle and screening of candidate mutation sites. (**A**) Luciferase assays were performed to test the transcriptional activity of different truncated promoters after H37Ra infection or no infection. Bovine *NRAMP1* promoters with different lengths and internal reference vectors were transfected into RAW264.7 cells for 48 h. The infected group was infected with H37Ra (MOI = 10) at 24 h after transfection. Values were normalized to the mean of the non-infected PGL4.10 group (±SD). Student’s *t*-test was used to evaluate the differences (*, *p <* 0.05). (**B**) A homology analysis of the *NRAMP1* promoter −278/+73 region across different species was conducted using DNAMAN. Cis-regulatory elements within this region were predicted by online software and those with JASPAR scores above the 75% threshold were identified. Elements within the 42% differential sequences of the *NRAMP1* promoters in mice and cattle were screened and classified using the online tool Venny 2.1.0. (bioinfogp.cnb.csic.es/tools/venny, accessed on 15 July 2022), and seven candidate sites were obtained, as indicated by the red circles that highlight the clustered candidate sites. (**C**) The effects of mutations at the seven candidate sites on promoter activity. The relative transcriptional activities of the promoters were determined by luciferase assays. The pGL4.1 vectors that contained the different promoter fragments were co-transfected with pRL-TK in RAW264.7. The H37Rainfected groups were infected with H37Ra (MOI = 10) 24 h after transfection and then incubated for 24 h. Values were normalized to the mean of the non-infected pGL4.1-T3(−278/+73) group (±SD). Student’s *t*-test was used to evaluate the differences (**, *p <* 0.01).

To detect and compare the core promoter regulating bovine *NRAMP1* expression after H37Ra infection, we progressively truncated the promoter fragment, analyzed transcriptional changes, and compared the relative transcriptional activities of the truncated promoters between the H37Ra-infected and non-infected groups. We generated pGL4.10 containing the sequence from −1611 to +73 bp (ARS-UCD 1.2 bovine assembly, relative to the TSS) and then progressively deleted the 5′ sequence of the *NRAMP1* promoter to generate eight truncated promoter reporter constructs, namely, pGL4.1-T1(−1242/+73), pGL4.1-T2(−792/+73), pGL4.1-T3(−278/+73), pGL4.1-T4(−264/+73), pGL4.1-T5(−214/+73), pGL4.1-T6(−162/+73), pGL4.1-T7(−104/+73), and pGL4.1-T8(−60/+73). pGL4.1-T3(−278/+73) exhibited sufficient activity compared to the other truncated promoters and demonstrated significantly higher activity after infection, indicating that this region is crucial for *NRAMP1* expression and may contain cis-regulatory elements responsive to H37Ra infection (Figure 1A).

Homology analysis revealed that the bovine *NRAMP1* promoter −278/+73 region is highly homologous to sheep, pigs, horses, and humans, while it diverges widely from the mouse *Nramp1* promoter (Figure 1B). We wanted to investigate whether sequence differences between cattle and mouse *Nramp1* promoters lead to variations in the recruitment of TB-associated TFs. To screen candidate TFs related to TB stimulation and their binding sites, cis-regulatory elements were predicted by P-Match, AliBaba2.1, SIGNAL SCAN (http://gene-regulation.com, accessed on 11 July 2022), and LASAGNA-Search 2 (https://biogrid-lasagna.engr.uconn.edu/, accessed on 11 July 2022, the website is currently unavailable), using the bovine *NRAMP1* promoter −278/+73 region or the homologous region in the mouse *Nramp1* promoter (−224/+176), respectively. Cis-regulatory elements for immune- and inflammatory-associated TFs were selected, and their sequences were scored using JASPAR (http://jaspar2016.genereg.net/, accessed on 11 July 2022). Those scoring above the 75% threshold were retained. Among the predicted cis-regulatory elements, those located within the differential sequence regions of bovine and mouse promoters were analyzed and clustered, leading to the identification of seven candidate sites for further investigation (Figure 1B).

### 2.2. Mutation at the 2SP Site Specifically Increases NRAMP1 Promoter Activity After H37Ra Infection

To determine the effects of the seven cis-regulatory elements in H37Ra infection, pGL4.1-T3(−278/+73) plasmids with mutations in each core motif were created, named POUM, EM,1SPM,2SPM,3SPM, etsM, and puM, respectively (Table S2). These seven recombinant plasmids were co-transfected with the internal reference vector pRL-TK into RAW264.7 cells, and the relative transcriptional activities were compared between the H37Ra-infected and non-infected groups. As shown in Figure 1C, 1SPM showed a significant decrease in *NRAMP1* promoter activity after H37Ra infection, whereas it only presented a faint decrease with no significant difference in the absence of H37Ra infection. Interestingly, after H37Ra infection, 2SPM led to a significant increase (2.3-fold) in *NRAMP1* promoter activity, but no difference was manifested in the non-infected group. On the other hand, 3SPM did not exhibit significant differences in *NRAMP1* expression in either the infected or the non-infected groups (Supplementary Figure S2A). POUM, EM, etsM, and puM all significantly reduced *NRAMP1* promoter activity, exhibiting similar magnitudes of reduction in both H37Ra-infected and non-infected groups (Supplementary Figure S2B–D).

### 2.3. Altering the Affinity of the 2SP Site for SP1/SP3 Affects NRAMP1 Promoter Activity After H37Ra Infection

We analyzed the homology of the bovine *NRAMP1* promoter with 108 species of ruminants and 32 other species using the Animal Omics Database (http://animal.omics.pro/, accessed on 5 August 2022). Bovine *NRAMP1* promoter was highly homologous to 130 of the total 140 species (Supplementary Figure S3). Further analysis of the homologous region of the 2SP site in 130 species revealed two primary conserved core motifs: 5′-GGGAGAGG-3′ (in cattle, sheep, and related species) and 5′-GGGAGG-3′ (in humans, pigs, and related species). These sequences were identified as variants of the consensus SP1/SP3 recognition sites. The TF-predicted scores from the JASPAR database suggest that the two variants exhibit weakened affinity for SP1/SP3 (7.18 and 4.83). However, six species lost affinity for SP1/SP3 due to mutations or deletions in the homologous region of 2SP site: they are gerenuks, horses, bats, hedgehogs, mice, and manatees.

**Figure 2:**
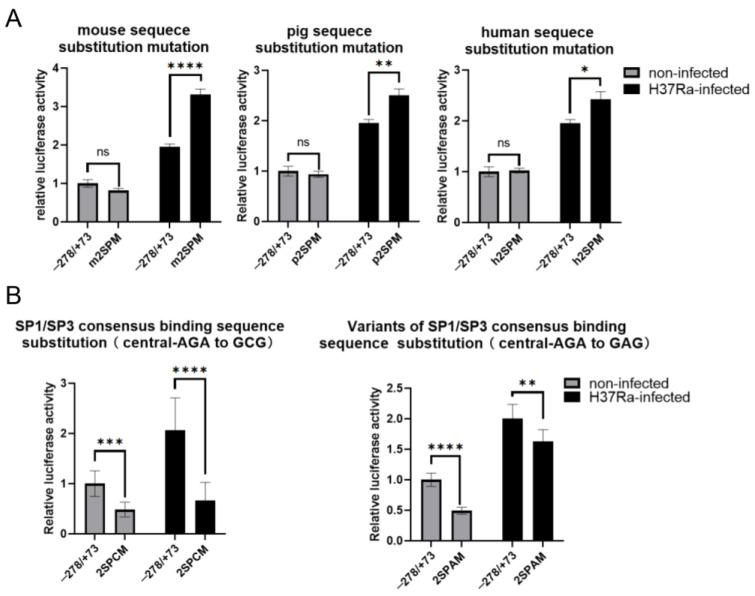
Altering the 2SP site affinity for SP1/SP3 affects *NRAMP1* promoter activity. (**A**) Mouse sequence substitution (no affinity for SP1/SP3) resulted in a substantial increase (1.7-fold) in *NRAMP1* promoter activity after H37Ra infection, while pig sequence substitution and human sequence substitution (weaker affinity for SP1/SP3) resulted in a moderate increase (1.28-fold and 1.24-fold, respectively) in *NRAMP1* promoter activity after H37Ra infection. The relative transcriptional activities of the promoters were determined by luciferase assays. Values were normalized to the mean of the non-infected pGL4.1-T3(−278/+73) group (±SD). Student’s *t*-test was used to evaluate the differences (ns, *p* ≥ 0.05; *, *p* < 0.05; **, *p* < 0.01; ****, *p* < 0.0001). Different mutated promoter activities in B were detected in the same way. (**B**) SP1/SP3 consensus binding sequence substitution resulted in a substantial decrease in *NRAMP1* promoter activity in both the H37Ra infection group (0.52-fold) and the non-infection group (0.28-fold), and repeated experiments using the THP-1 cell line yielded consistent results (0.48-fold and 0.32-fold, respectively). The variants of SP1/SP3 consensus binding sequence substitution resulted in a moderate decrease in *NRAMP1* promoter activity in both the H37Ra infection group (0.85-fold) and the non-infection group (0.49-fold) (**, *p* < 0.01; ***, *p* < 0.001; ****, *p* < 0.0001).

Next, we aimed to determine whether the altered SP1/SP3 binding affinity at the 2SP site was a contributing factor to the changes in bovine *NRAMP1* promoter activity after H37Ra infection. We first investigated the effect of eliminating or weakening SP1/SP3 binding at the 2SP site on *NRAMP1* expression. Sequence analysis revealed that the region corresponding to the 2SP site in the mouse *Nramp1* promoter lacks SP1/SP3 binding affinity, while equivalent regions in human and pig promoters exhibit relatively weaker affinity compared to cattle. We introduced mutations into pGL4.1-T3(−278/+73) using the sequences corresponding to the 2SP site from mice, humans, and pigs, designated as m2SPM, h2SPM, and p2SPM, respectively. Promoter activities were assessed under both H37Ra-infected and uninfected conditions. In H37Ra-infected groups, substituting the 2SP site with the corresponding mouse sequence resulted in a substantial increase in promoter activity (1.7-fold), whereas substitution with the human and pig sequences also significantly increased promoter activity, but to a lesser extent (1.28-fold and 1.24-fold). On the other hand, these three mutated promoters showed no significantly different activities to pGL4.1-T3(−278/+73) in the non-infected group (Figure 2A). To further evaluate the reliability of the differences in SP1/SP3 binding affinity for the 2SP site across different species, 3D structural models of bovine SP1 and SP3 were constructed, and molecular docking simulations were performed. Molecular docking provides a computational method for predicting the binding modes and affinities of protein–ligand interactions, which can help elucidate the structural basis of species-specific differences in binding behavior. The 3D model structures of SP1 and SP3 proteins, along with their predicted binding pockets, showed a high degree of similarity (Table 1). Additionally, both proteins exhibited high average conservation scores, indicating their evolutionary conservation. These findings align with previous reports suggesting that SP1 and SP3 recognize identical binding sites and are conserved across species [35,36,37]. Using bovine SP1 as an example, we observed that the High Ambiguity Driven DOCKing (HADDOCK) scores of the 2SP site sequences from cattle, humans, pigs, and mice increased progressively, indicating a corresponding decrease in SP1 binding affinity. Consistently, replacing the bovine 2SP site sequence with those from humans, pigs, and mice resulted in a corresponding increase in promoter activity following H37Ra infection. This finding supports the relationship between 2SP site sequence variation and its functional impact on promoter activity.

Conversely, we investigated the impact of enhanced SP1/SP3 binding affinity at the 2SP site on *NRAMP1* promoter activity. Previous studies have reported that the consensus SP1-binding sequence is GGGGCGGGG, and SP3 shares structural similarities with SP1, resulting in similar affinities for the SP1-binding site [38]. The central A-substituted variant (GGGGAGGG) represents a weaker affinity variant of the consensus SP1/SP3 recognition site [39]. To test whether a stronger SP1/SP3 binding affinity at the 2SP site would alter bovine *NRAMP1* promoter activity, we mutated the 2SP site to 5′-GGGGAGGG-3′ (2SPAM) and 5′-GGGGCGGG-3′ (2SPCM), which exhibited a stronger affinity and the strongest affinity for SP1/SP3, respectively. Both 2SPCM and 2SPAM mutations significantly decreased bovine *NRAMP1* promoter activity in both H37Ra-infected and non-infected groups, with 2SPCM showing a more pronounced reduction (0.32-fold) compared to 2SPAM (0.82-fold) in the H37Ra-infected group (Figure 2B).

We also generated pGL4.10 containing the mouse *Nramp1* promoter sequence from −1611 bp to +156 bp. In accordance with the sequences of bovine and humans, the mouse homologous region sequence of the 2SP site was substituted to 5′-GGGAGAGG-3′ and 5′-GGGAGG-3′, namely, MbM and MhM, respectively. Luciferase assays revealed that the two substitutions significantly reduced promoter activity in the H37Ra-infected group; however, no significant difference was manifested in the non-infected group (Supplementary Figure S4).

### 2.4. SP1 and SP3 Play Different Roles in Regulating the Activity of the Nramp1 Promoter

SP1 is a key regulator of myeloid-specific genes including *NRAMP1* [40]. SP1 and SP3 are members of the SP (specificity protein) TF family; they are structurally similar and have similar affinities for the same binding site [38]. The highly conserved protein structure of SP1 and SP3 in mammals suggests a similar role in regulating *NRAMP1* expression in different mammals.

**Figure 3:**
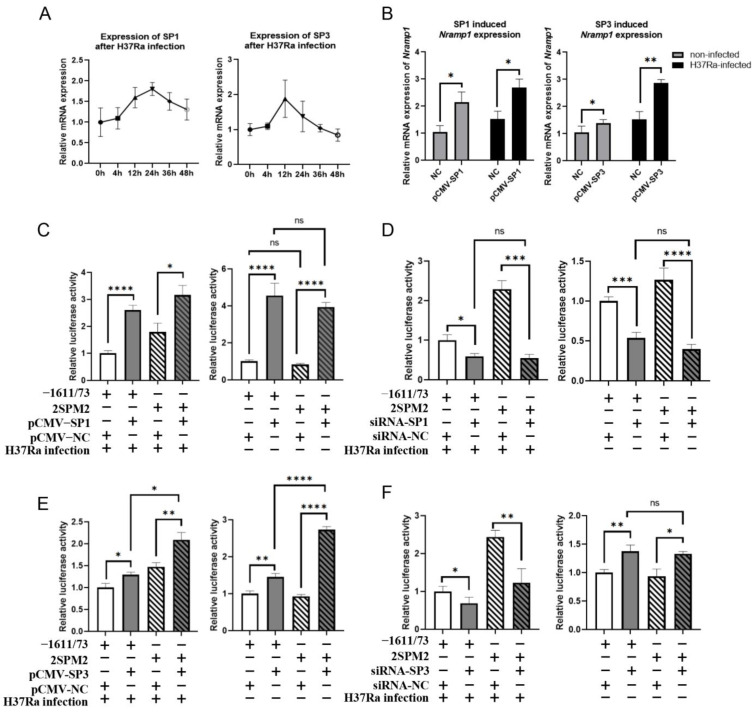
The roles of SP1 and SP3 in regulating *NRAMP1* expression and the impact of the 2SP site mutations on SP1 or SP3’s regulatory effects. (**A**) The expression levels of *Sp1* and *Sp3* in response to H37Ra infection. RAW264.7 cells were infected with H37Ra (MOI = 10) for 0 h (uninfected), 4 h, 12 h, 24 h, 36 h, and 48 h. The expressions of *Sp1* and *Sp3* were detected by qPCR. (**B**) The regulation of SP1 or SP3 overexpression on *Nramp1* expression in H37Ra-infected/non-infected cells, respectively. pCMV-SP1, pCMV-SP3, and NC groups were transfected into RAW264.7 cells after H37Ra infection or no infection. The infected group cells were infected with H37Ra (MOI = 10) at 24 h after transfection. The expressions of *Nramp1* were detected by qPCR (*, *p* < 0.05; **, *p* < 0.01). (**C**,**D**) The impact of the 2SPM2 mutation on SP1 overexpression or knockdown in H37Ra-infected and non-infected groups, respectively. On the left side is the H37Ra-infected group, and on the right side is the non-infected group. The white and dark columns represent the blank control and the overexpression/knockout treatment, respectively. The diagonally hatched columns indicate that the promoter used in this group are the 2SPM2 mutant promoter. In (**E**,**F**), the representations are consistent. Values were normalized to the mean of the −1611/+73 + pCMV group (ns, *p* ≥ 0.05; *, *p* < 0.05; ***, *p* < 0.001; ****, *p* < 0.0001). (**E**,**F**) The impact of the 2SPM2 mutation on SP3 overexpression or knockdown in H37Ra-infected and non-infected groups, respectively. Values were normalized to the mean of the −1611/+73 + pCMV group (ns, *p* ≥ 0.05; *, *p* < 0.05; **, *p* < 0.01; ****, *p* < 0.0001).

To elucidate how the altered binding affinity of SP1/SP3 at the 2SP site specifically enhances *NRAMP1* promoter activity after H37Ra infection, we aimed to investigate the roles of SP1 and SP3 in this process. We first detected the expression levels of Sp1 and Sp3 in RAW264.7 cells following H37Ra infection. As shown in Figure 3A, *Sp1* expression peaked at 24 hpi and subsequently declined, returning to near baseline levels by 48 hpi. *Sp3* expression peaked at 12 hpi and returned to baseline by 36 hpi.

Although the mechanism is not fully elucidated, both SP1 and SP3 are ubiquitously expressed factors but can regulate specific gene expression. One explanation is that SP3 is a weaker transcriptional activator than SP1, and they compete for binding to the same binding site; therefore, the ratio of SP1:SP3 proteins may affect target gene expression [41,42]. Another study found that both SP1 and SP3 can regulate the basal expression of target genes, but only SP3 participates in the IFN-inducible complex formation with other TFs, including STAT1, STAT2, and IRF9, at the target gene promoter to regulate specific gene expression [43]. To further explore whether SP1/SP3 and several other potential candidate TFs (STAT1, STAT3, IRF1, IRF2, IRF3, and IRF9) are involved in the specific gene expression of *Nramp1* induced by H37Ra, the overexpression recombinant plasmids pCMV-SP1, pCMV-SP3, pCMV-STAT1, pCMV-STAT3, pCMV-IRF1, pCMV-IRF2, pCMV-IRF3, or pCMV-IRF9 were transfected into RAW264.7 cells and infected with H37Ra or not. The results showed that in the non-infected group, SP1, SP3, IRF1, IRF3, and STAT1 significantly enhanced *Nramp1* expression, while in the H37Ra-infected group, SP1, SP3, IRF2, and IRF3 significantly increased *Nramp1* expression (Supplementary Figure S5). It is worth noting that in the non-infected group, SP3 overexpression slightly upregulated *Nramp1* expression (1.3-fold), whereas after H37Ra infection, it markedly enhanced the activation of *Nramp1* expression (1.9-fold). However, SP1 overexpression exhibited similar activation ability (1.8-fold and 2.0-fold) regardless of infection status (Figure 3B).

### 2.5. Validating the Impact of the 2SP Site Mutations on the SP1/SP3-Mediated Regulation of NRAMP1 Expression

Previous studies have demonstrated that for promoters containing multiple Sp-binding sites, Sp1 may bind at both proximal and distal transcriptional sites to exert its transcriptional synergism [38,44,45]. According to the prediction, the bovine *NRAMP1* promoter contains multiple SP-binding sites. To more accurately assess the impact of 2SP site mutations, we utilized the pGL4.10 vector (pGL4.10−1611/+73) containing the bovine *NRAMP1* promoter sequence from −1611 to +73 bp for the functional validation of the 2SP site mutations in subsequent experiments. To minimize potential adverse effects and enhance safety, we aimed to reduce the number of mutated bases at the 2SP site. Two editing sequences that reduced the number of mutant bases were designed, along with a complete deletion of the 2SP site as a comparison, namely, PGL4.10-2SPM1, PGL4.10-2SPM2, and PGL4.10-2SPM3, respectively. Both 2SPM1 and 2SPM2 mutations significantly increased promoter activity after H37Ra infection. However, the 2SPM2 mutant promoter exhibited a more similar activity to the −1611/+73 promoter in the uninfected group (Supplementary Figure S6). Consequently, the 2SPM2 mutation (5′-GGGAGAGGTG-3′ to 5′-AAAAGAGGTG-3′) was selected for further investigation.

To investigate the impact of the 2SPM2 mutation on the SP1/SP3-mediated regulation of bovine *NRAMP1* expression, the overexpression recombinant plasmid of SP1/SP3 or siRNA against SP1/SP3 was co-transfected with PGL4.10-2SPM2 or pGL4.10−1611/+73 in RAW264.7 cells. The results indicated that the overexpression of SP1 significantly increased the activity of the −1611/+73 promoter and the 2SPM2 mutant promoter in both H37Ra-infected and non-infected cells. In non-infected cells, there was no significant difference in promoter activity between the −1611/+73 promoter and the 2SPM2 mutant promoter, regardless of SP1 overexpression (Figure 3C). The knockdown of SP1 significantly decreased −1611/+73 promoter and 2SPM2 mutant promoter activities in both H37Ra-infected and non-infected cells. In both the infected and non-infected cells, there was no significant difference in the activity of the 2SPM2 mutant promoter and the −1611/+73 promoter following SP1 knockdown (Figure 3D). Based on the previous results, after H37Ra infection, SP1 expression was upregulated, leading to the activation of *NRAMP1* expression. In molecular docking, the HADDOCK score of 2SPM2 increased from −88.9 to −37.5 upon mutation, which suggests a marked reduction in its binding affinity. Collectively, these findings suggest that the 2SPM2 mutation does not impair the ability of SP1 to activate *NRAMP1* expression.

The overexpression of SP3 significantly increased the activities of the −1611/+73 promoter and the 2SPM2 mutant promoter in both H37Ra-infected and non-infected cells. Following SP3 overexpression, the upregulation was greater in the 2SPM2 mutant promoter (1.42-fold in infected and 2.95-fold in non-infected cells) compared to the −1611/+73 promoter (1.29-fold in infected and 1.45-fold in non-infected cells) (Figure 3E). The knockdown of SP3 significantly decreased the activities of both promoters in H37Ra-infected cells (to 0.68-fold for −1611/+73 and 0.5-fold for 2SPM2), while in non-infected cells, SP3 knockdown resulted in increased promoter activities (Figure 3F). Based on the previous results, after H37Ra infection, SP3 expression is upregulated, and its ability to activate *NRAMP1* expression is also enhanced. These results indicate that following H37Ra infection, the 2SPM2 mutant promoter exhibits increased sensitivity to SP3 regulation. On the other hand, in non-infected cells, although SP3 overexpression slightly increases *NRAMP1* expression, its activating capacity is weaker compared to SP1. SP3 competes with SP1 for binding to the same regulatory site, potentially inhibiting SP1’s activation. Consequently, SP3 knockdown results in enhanced *NRAMP1* expression.

### 2.6. Base Editing of the Homologous Region of the 2SP Site in THP-1 Cells and Phenotypic Verification

**Figure 4:**
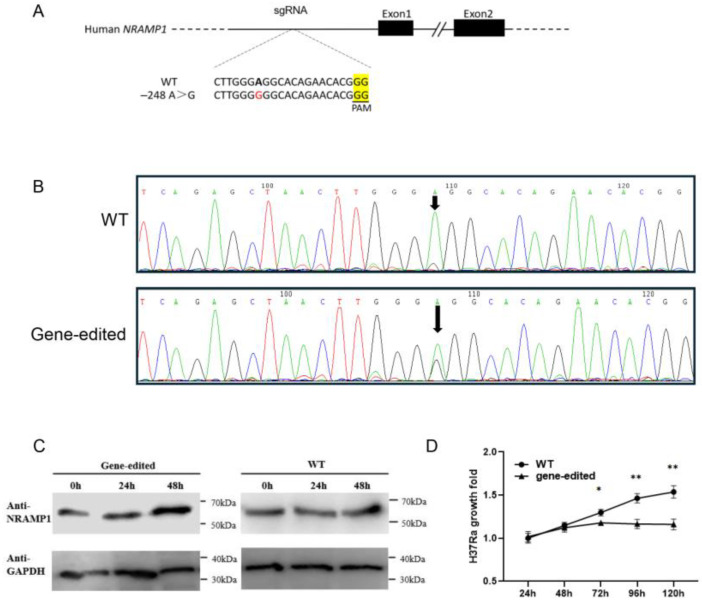
Base editing of the homologous region of the 2SP site in THP-1 cells and phenotypic verification. (**A**) The sgRNA designed for editing the homologous region of the 2SP site in THP-1 cells. The bolded A denotes the target site for editing in the wild-type sequence, whereas the red-highlighted G signifies the mutated base being G, and the yellow highlighting indicates the location of the PAM site. (**B**) The sequence of the *NRAMP1* promoter in the edited clone. The edited site is indicated by the arrow. The nucleotide sequence before and after the edited site was identical to the wild type. (**C**) Western blot analyses to detect NRAMP1 expression in gene-edited cells and wild-type THP-1 cells after H37Ra infection. (**D**) Multiplication of H37Ra in wild-type THP-1 cells or gene-edited cells. The values of the gene-edited groups and the wild-type group at 48 h, 72 h, 96 h, and 120 h were normalized to the mean of the wild-type group at 24 h (±SD). Student’s *t*-test was used to evaluate the differences (*, *p* < 0.05; **, *p* < 0.01).

To verify the endogenous expression of *NRAMP1* after editing, we managed to generate macrophage cell lines with the 2SP site edited. Due to the lack of bovine macrophage cell lines and the high homology of bovine and human *NRAMP1* promoters, a base editing system for the 2SP mutation of the THP-1 cell line was designed and constructed. A previous study proved that a central G-substitution at the Sp1/Sp3 recognition site would reduce binding affinity by at least 30-fold; thus, the guide was designed to make a central A-to-G mutation in the homologous region of the 2SP site. The epi-ABEmax-NG-h2SPM vector, containing the designed sgRNA, was constructed based on epi-ABE4max-NG (Addgene plasmid #135976) for THP-1 cell editing, with the aim of introducing a central A-to-G mutation (5′-GGGAGG-3′ to 5′-GGGGGG-3′) (Figure 4A). The frequencies of precisely edited cells were detected by sequencing the mixed cell population. Under several different experimental systems we tested, the frequency of precisely edited cells reached a maximum of 0.33% (Supplementary Figure S7). Out of twenty-five 96-well plates, a total of 272 clones survived the sorting process, and 1 clone was confirmed to be precisely edited as a heterozygote with the expected single nucleotide mutation (A to G) at the target site. The sequence is shown in Figure 4B.

The production of the NRAMP1 protein in the gene-edited clones and wild-type THP-1 cells before/after H37Ra infection was assessed by Western blotting. Before H37Ra infection, the gene-edited group exhibited comparable NRAMP1 expression levels to the wild-type group; however, as the duration of infection progressed, NRAMP1 expression in the gene-edited group increased more remarkably compared to the wild-type group (Figure 4C). To further evaluate the potential of gene-edited THP-1 cells in enhancing the inhibition of Mtb proliferation, CFU counts were enumerated at 24, 48, 72, 96, and 120 h post-H37Ra infection. The gene-editing group exhibited a significantly enhanced inhibition of H37Ra multiplication compared to those in the wild-type group after 72h (Figure 4D).

## 3. Discussion

In this paper, we describe the screening of bovine *NRAMP1* promoter mutation site gene editing to enhance TB resistance. A novel mutation site designated as 2SP was identified. The mutation of this site specifically upregulates the activity of the bovine *NRAMP1* promoter after H37Ra infection while it does not affect basal expression in uninfected groups. Subsequently, THP-1 cell lines with the homologous region of the 2SP site edited were generated using the CRISPR-Cas9 base editing technique. In fact, we also established a screening system for bovine fetal fibroblasts (BFFs) with 2SP site editing and obtained correct edited BFFs. These BFFs are used to produce edited-gene cattle.

*NRAMP1* has been shown to be critical for host resistance to TB and a previous study in our laboratory demonstrated that gene-edited *NRAMP1* overexpression cattle increased TB resistance [33,34]. However, the insertion of synthetic sequences still poses potential security risks. Thus, the aim of this study was to specifically upregulate bovine *NRAMP1* expression after TB infection through endogenous mechanisms, while minimizing its impact on basal transcription in the absence of infection.

Studies have shown that *NRAMP1* is evolutionarily conserved, indicating that its structure and function have remained stable and sufficient throughout evolution. Mutations in its structure are more likely to be harmful. However, mutations in non-coding regions that alter the expression rather than the protein structure may be beneficial for TB resistance [18,30]. On the other hand, despite the conserved structure of *NRAMP1* being shared among different species, there are variations in their TB resistance. For example, certain strains of mice are considered to be resistant to TB [46,47], while most hoofed stock are considered to be susceptible [48]. This variation could be associated with different expression levels of *NRAMP1*. To test this hypothesis, we compared the levels of TB-induced *NRAMP1* gene expression between cows and mice using the data uploaded from different labs. As expected, mice showed stronger TB-induced *Nramp1* upregulation than cows, both in vivo and in vitro.

In eukaryotes, gene transcription is controlled by fundamental transcriptional mechanisms consisting of TFs and various sequence motifs that interact with basal TFs in the core promoter regions [49]. Recently, with the development of gene editing technologies, base editing has been used to generate or revert mutations in core promoter regulatory regions, aiming to regulate the expression of target genes [50,51,52]. These edits typically occur at frequently mutated sites within core promoter regions, and the efficient editing of key sites is critical for the success of this technique. However, studies on natural mutations in the bovine *NRAMP1* promoter associated with TB resistance remain insufficient, and no mutation editing site within the *NRAMP1* promoter has been reported to enhance TB resistance. In this study, we aimed to identify and screen potential mutation editing sites using interspecies sequence differences in the *NRAMP1* promoter and predicted TF binding sites as references.

Among the screened candidate mutation sites, the 2SP site showed great potential for mutation editing. The homologous region of the 2SP site exhibited two conserved sequences in 124 out of the total 130 species with *NRAMP1* gene promoter homology, which were variants of the consensus SP1/SP3 recognition site. Six species lost affinity for SP1/SP3 due to mutations or deletions in the homologous region of the 2SP site. These include mice and horses, with some strains exhibiting relative resistance to TB [47,53], as well as bats, which are thought to have strong immune system [54,55], implying that the loss of affinity for SP1/SP3 at the 2SP site may benefit to TB resistance. By substituting the 2SP site with corresponding sequences from humans, pigs, and mice and predicting their respective affinities for SP1/SP3, we observed that a lower affinity of the 2SP site correlates with higher promoter activity following H37Ra infection. In contrast, replacing the 2SP site with conserved SP1/SP3 binding sequences significantly reduced promoter activity, with a more pronounced reduction observed post-infection. In a study investigating the SNP within the SP1/SP3 binding site of the CD14 promoter, it was found that this SNP reduces the affinity of the binding site while enhancing promoter activity. The authors further suggested that this effect might be attributed to the decreased affinity of the binding site for SP3 [56].

In this study, we found that the overexpression of both SP1 and SP3 activates bovine *NRAMP1* basal transcription. SP1 demonstrated a greater ability to activate basal transcription, while only SP3, not SP1, specifically enhanced its ability to activate *NRAMP1* expression after H37Ra infection. We speculate that only SP3 forms an Mtb-induced complex with other TFs to activate *NRAMP1* expression during infection specifically. This is consistent with a previous study that suggested that SP1 plays a role in basal transcription, while SP3 is involved in inducible transcription [43]. However, further experiments are still needed to verify this assumption.

Except for the 2SP site, mutations at the 1SP site (−262/−253bp), an adjacent upstream SP1/SP3 recognition site relative to the 2SP site, also showed a specific response to H37Ra infection, but with the opposite effect. Specifically, the 1SP site mutation did not alter promoter activity in uninfected conditions but significantly reduced promoter activity after infection. These imply that the 1SP site may be a “correct” recognition site for the specific regulation of SP3; however, the 2SP site may not be a “correct” recognition site, and its weak affinity makes it compete with the 1SP site to bind SP3.

Therefore, the mechanism through which the 2SP site mutation can specifically upregulate *NRAMP1* expression after infection but not affect its expression in uninfected conditions may be as follows: in uninfected conditions, SP1 plays a dominant role in activating *NRAMP1* expression, and its activation is independent of binding to the 1SP site or the 2SP site. Consequently, mutation at the 2SP site does not affect the basal expression of *NRAMP1*. During H37Ra infection, SP3 specifically activates *NRAMP1* expression by binding to the 1SP site. However, the adjacent 2SP site competes for SP3 binding, thereby inhibiting this activation. Therefore, mutations at the 2SP site reduce competition for SP3 binding, enhance the binding efficiency of SP3 at the 1SP site, and thus specifically upregulate *NRAMP1* expression during infection.

Finally, we established a base-editing screening system targeting the homologous region of the 2SP site in THP-1 cells and successfully generated THP-1 cells with an edited 2SP site. Upon validation, the edited cells exhibited significantly elevated NRAMP1 expression levels following H37Ra infection and effectively suppressed bacterial proliferation.

In summary, through interspecific comparison and sequence analysis, we identified for the first time a potential editing site in the bovine *NRAMP1* promoter that may specifically upregulate *NRAMP1* expression in response to Mtb infection. Subsequently, we generated THP-1 cell lines with the homologous region of the 2SP site edited, which exhibited enhanced TB resistance. Moreover, BFFs with the 2SP site edited were also generated and used for nuclear transfer. We will further optimize the experimental system to obtain a sufficient number of precisely edited BFFs for screening high-quality clones suitable for nuclear transfer. Consequently, a limitation of this study is that the findings were not verified in vivo, which will be addressed in subsequent experiments.

## 4. Materials and Methods

### 4.1. qPCR

Total RNA was isolated using TransZol reagent (TransGen, Beijing, China) and subsequently transcribed into cDNA using EasyScript One-Step RT-PCR SuperMix (TransGen, Beijing, China). The quantitative primers of mouse *Nramp1*, mouse *Sp1*, mouse *Sp3*, and human *NRAMP1* were designed, and mouse glyceraldehyde 3-phosphate dehydrogenase (*Gapdh*) and human *Gapdh* were selected as normalization controls, respectively (Table S1). The PCR cycling conditions were performed according to the manufacturer’s protocols for TransStart FastPfu DNA Polymerase (TransGen, Beijing, China).

### 4.2. Construction of Vectors

The bovine *NRAMP1* promoter reporter vector pGL4.10 −1611/+73 and mouse *Nramp1* promoter reporter vector pGL4.10 m−1611/+156 were generated by inserting the bovine promoter fragment and mouse promoter fragment into the luciferase reporter vector pGL4.10, respectively. The truncated bovine *NRAMP1* promoter reporter vectors pGL4.1-T1(−1242/+73), pGL4.1-T2(−792/+73), pGL4.1-T3(−278/+73), pGL4.1-T4(−264/+73), pGL4.1-T5(−214/+73), pGL4.1-T6(−162/+73), pGL4.1-T7(−104/+73), and pGL4.1-T8(−60/+73) were constructed based on the pGL4.10 −1611/+73. The primers are shown in Table S2.

The 7 potential TF binding site mutations for the bovine *NRAMP1* promoter fragment (POUM, EM,1SPM,2SPM,3SPM, etsM, 2SPM1, 2SPM2, and 2SPM3) and the homologous regions of 2SP site substitution/deletion for the mouse *Nramp1* promoter fragment (MbM and MhM) were generated by overlap PCR or directly using the primers contain the mutation. The primers are shown in Table S3. Then, the fragments were inserted into the luciferase reporter vector pGL4.10.

To verify the effects of optimized mutations at the 2SP locus, PGL4.10 vectors harboring the 2SPM1, 2SPM2, and 2SPM3 mutations were constructed using an overlap extension method (Table S4).

In the overexpression experiments, cDNA extracted from RAW264.7 cells or BFF was used as a template to amplify the coding region sequence of the target gene. The amplified sequences were then subcloned into the pCMV vector (Takara, Beijing, China). An empty pCMV vector served as the negative control. In the knockdown experiments, specific siRNAs against targets SP1 and SP3 and negative control siRNA were synthesized from Genepharma.

epi-ABEmax-NG-h2SPM was generated based on epi-ABE4max-NG (Addgene plasmid #135976) [57]. The guide was designed to target the homologous region of the 2SP site in the human *NRAMP1* promoter (5′-CTTGGGAGGCACAGAACACG-3′), aiming to produce a central A to G mutation (5′-GGGAGG-3′ to 5′-GGGGGG-3′).

### 4.3. Cell Culture

RAW264.7 cells, obtained from the American Type Culture Collection, were cultured in Dulbecco’s modified Eagle’s medium (Gibco, Waltham, MA, USA) containing 10% fetal bovine serum (Gibco, Waltham, MA, USA) at 37 °C in a 5% CO_2_ environment.

THP-1 cells, obtained from the American Type Culture Collection, were cultured in RPMI1640 medium (Gibco, Waltham, MA, USA) containing 10% fetal bovine serum (Gibco, Waltham, MA, USA) at 37 °C in a 5% CO_2_ environment.

### 4.4. H37Ra Infection

H37Ra strains were cultured in 7H9 media (Becton Dickinson, Franklin Lakes, NJ, USA) supplemented with 10% OADC at 37 °C in a 5% CO_2_ environment to logarithmic phase. Before infection, the final concentration of bacteria was calculated. At 24 h post-transfection, the appropriate number of bacteria were separated from the stock culture, centrifuged at 12,000× *g* for 1 min, and resuspended in RAW264.7 or THP-1 media for MOI = 10. Then, the bacteria were added to RAW264.7 cells or THP-1 cells for 4 h at 37 °C, 5% CO_2_, before being washed twice with PBS to remove extracellular bacteria, and were then cultured in fresh media containing 10% fetal bovine serum.

### 4.5. Luciferase Assays

RAW264.7 cells were seeded in 24-well plates one day before transfection and grown to a confluence of 60%. Then, 0.5 μg of pGL4.10-promoter or empty vector pGL4.10 and 0.1 μg of pRL-TK normalizing vector were co-transfected according to the protocol of the PEI Transfection Reagent (MedChemExpress, Monmouth Junction, NJ, USA).

In total, 1 × 10^6^ THP-1 cells per sample were centrifuged at 1050 rpm for 5 min, resuspended in RPMI1640 without fetal bovine serum, and seeded in a 24-well plate one day before transfection. Then, 0.5 μg of pGL4.10-promoter or empty vector pGL4.10 and 0.1 μg of pRL-TK normalizing vector were co-transfected according to the protocol of the PEI Transfection Reagent (MedChemExpress, Monmouth Junction, NJ, USA).

At 48h post-transfection, RAW264.7 or THP-1 cell lysates were collected for luciferase activity analysis by using the Double-Luciferase Reporter Assay Kit (TransGen, Beijing, China).

### 4.6. Editing of NRAMP1 in THP-1 Cells

In this study, we performed a central A to G mutation in the homologous region of the 2SP site in the *NRAMP1* promoter (−248A to G) with THP-1 cells, to mimic the editing at the 2SP site in the bovine *NRAMP1* promoter and validate the expression level. The human *NRAMP1* promoter sequences containing the homologous region of the 2SP site from −30 bp upstream to +30 bp downstream were entered into the base editing online design software BE-Designer (http://www.rgenome.net/be-designer/, accessed on 4 October 2022) [58], selecting ‘5′-NG-3′’ for PAM type, ‘Homo sapiens (GRCh38/hg38) -Human’ for target genome, ‘ABE (A to G)’ for base editing type, and ‘14–17’ for window, and outputting the results. SgRNA selection was performed using an evaluation model as described in Reference [34]. Three sgRNAs were obtained and input into Cas-OFFinder (http://www.rgenome.net/cas-offinder/, accessed on 4 October 2022) [59] to select the sgRNA with fewer potential off-target sites (the option “Mismatch Number (equal to or less than)” was, respectively, set to 3 and 5). The detailed data are provided in Table S5. The selected sequence was 5′-CTTGGGAGGCACAGAACACG-3′.

The epi-ABEmax-NG-h2SPM vector containing the designed sgRNA was generated for THP-1 cell editing. The transfection of epi-ABEmax-NG-h2SPM into THP-1 cells was performed using the same method and proportion of plasmid as that used in the luciferase assays. Five days after transfection, 20 μg/mL of blasticidin was used for screening. The screening lasted 2 days before diluting into 10 cells/mL and seeding in 96-well plates. The number of cells in each well was examined under a microscope and the wells with a single cell were marked and left to grow for 2 weeks or more. When the number was sufficient, half of the cells from each marked well were used for positive identification, and the correct clones were kept for further experimentation.

### 4.7. Western Blot Analyses

Cells were lysed in ice-cold RIPA cell buffer containing protease inhibitors (Thermo Scientific, Waltham, MA, USA). The proteins were separated with 12% acrylamide gels and subsequently transferred to PVDF membranes (Millipore, Burlington, MA, USA). The primary antibodies used to detect NRAMP1 (Rockville, Dubai, United Arab Emirates, Catalogue No. ab59696) and GAPDH (Cell Signaling Technology, Danvers, MA, USA, Cat# 2118L) were diluted 1:500. Goat anti-rabbit IgG (zhuangzhibio, Xi’an, China, EK020) was used as secondary antibody for all primary antibodies, diluted 1:5000.

### 4.8. SP1/SP3 3D Structure Modeling and Ligand Docking

The SP1 and SP3 protein structure modeling was carried out in AlphaFold3 [60]. The dsDNA secondary structures of the 2SP site sequences from different species and the mutated sequences were predicted using the Sequence to Structure web tool, which is accessible at the following URL (https://scfbio-iitd.res.in/software/drugdesign/bdna.jsp, accessed on 27 March 2025). Functional domain was identified using the InterPro tool (https://www.ebi.ac.uk/interpro/, accessed on 27 March 2025) [61], and P2Rank (https://prankweb.cz/, accessed on 27 March 2025) [62] was used for predicting, visualizing, and scoring the binding pocket. The docking of ligand molecules (DNA) was performed and scored in Haddock [63,64]

## Figures and Tables

**Table 1:** Prediction of binding pockets in bovine SP1/SP3-modeled protein and corresponding scoring and parameters of the predicted binding pockets (top). The energy of interaction between bovine SP1-modeled protein and ligands: dsDNA sequences of the 2SP site in different species and the mutant sequence used for gene editing (bottom).

	Binding Pocket Prediction							
P2Rank	Bovine SP1 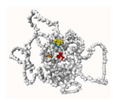				Bovine SP3 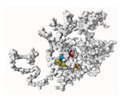			
Rank *	1	2	3	4	1	2	3	4
Score	5.72	4.11	2.33	2.00	7.14	5.23	3.65	1.09
Probability	0.245	0.146	0.049	0.036	0.341	0.215	0.064	0.007
Number of residues	9	8	5	5	10	12	6	6
Average conservation score	78.637	84.98	80.862	88.962	77.02	80.285	81.635	65.845
Energy of interaction (docking with bovine SP1)								
	2SP (cattle)	h2SPM (humans)		p2SPM (pigs)		m2SPM (mice)		2SPM2 (mutation)
HADDOCK score **	−88.9 ± 14.3	−72.0 ± 15.8		−65.5 ± 10.4		−64.8 ± 13.5		−37.5 ± 7.9
Van der Waals energy	−91.4 ± 6.2	−72.5 ± 12.0		−80.1 ± 9.1		−74.7 ± 16.7		−70.4 ± 3.8
Electrostatic energy	−379.3± 35.3	−335.1 ± 32.6		−198.2 ± 46.8		−215.1 ± 28.9		−272.3 ± 4.3
Desolvation energy	32.9 ± 2.5	28.0 ± 2.3		17.5 ± 1.7		18.9 ± 1.4		27.5 ± 1.3
Sum of energies	−526.7	−451.6		−326.3		−335.6		−352.7
Buried surface area	2487.8 ± 121.2	2114.5 ± 303.0		1881.0 ± 238.7		1930.4 ± 319.8		2019.9 ± 96.7

* Ranks of different colors correspond to pockets of the same color in the 3D structure. ** A composite energy metric combining van der Waals, electrostatic, and desolvation contributions; lower scores indicate stronger binding.

## Data Availability

The data supporting the findings of this study are available from the corresponding author upon reasonable request. These data encompass all relevant experimental results and analyses and will be provided to researchers who wish to verify or extend this work.

## Supplementary Materials

The following supporting information can be downloaded at: https://www.mdpi.com/article/10.3390/ijms26094229/s1. The Supplementary Materials have cited the following references [65,66,67,68,69].

## Abbreviations

The following abbreviations are used in this manuscript:

TB	tuberculosis
Mtb	*Mycobacterium tuberculosis*
*M. bovis*	*Mycobacterium bovis*
NRAMP1	natural resistance-associated macrophage 1
MAF	minor allele frequency
TF	transcription factor
*Gapdh*	glyceraldehyde 3-phosphate dehydrogenase
MOI	multiplicity of infection
hpi	hours post-infection
HADDOCK	High Ambiguity Driven DOCKing
SP	specificity protein
BFFs	bovine fetal fibroblasts
