# Supplementary material for: The 2SP Site Mutation in the Bovine Natural Resistance-Associated Macrophage 1 Promoter Exhibits Antituberculosis Potential
Source: Int J Mol Sci. 2025 Apr 29;26(9):4229. doi: 10.3390/ijms26094229 (PMC12071736; doi:10.3390/ijms26094229)
Supplement: Supplementary file 1 [file ijms-26-04229-s001.zip › ijms-3515185-supplementary.pdf]

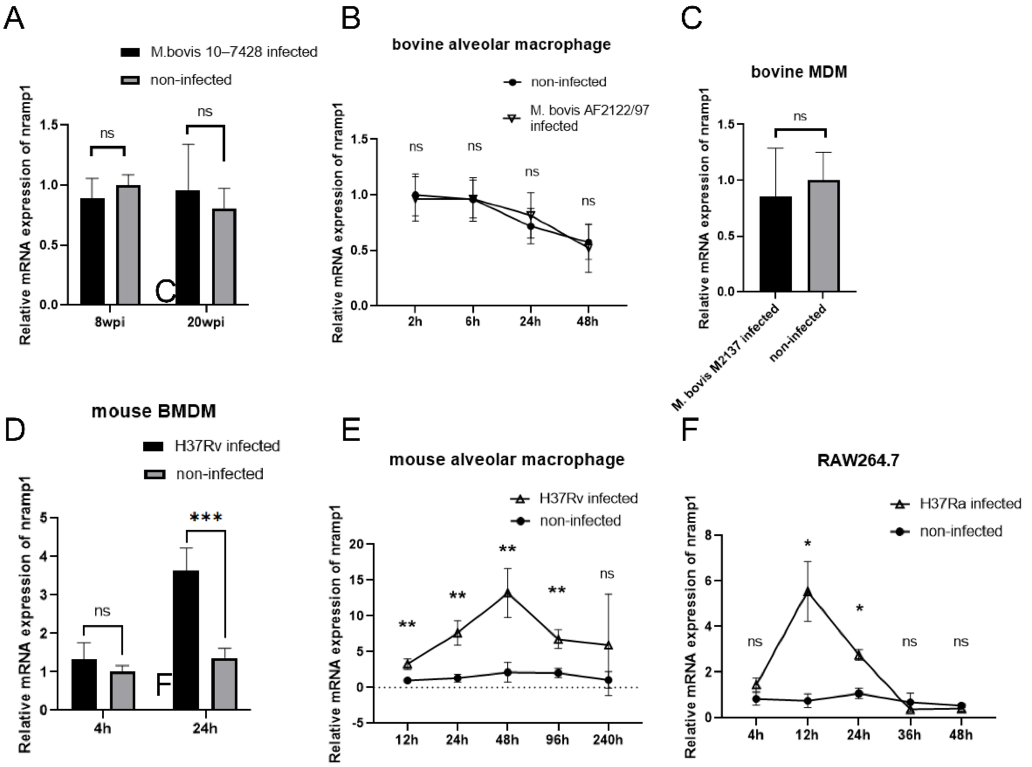

Supplementary Figure S1. The *NRAMP1* avtivation by Mtb infection in cattle and mice. (A) The avtivation of bovine *NRAMP1* in whole blood cells of Holstein cows by aerosol of M.bovis strain10-7428 infection is limited in early stage (8wpi) and late stage (20wpi) in vivo. The values of the M.bovis infected groups and non-infected group (20wpi) were normalized to the mean of the non-infected group(8wpi) (±SD). Student's *t*-test was used to evaluate the differences.The GEO file number is GSE192537 [65] (B)The activation of bovine *NRAMP1* in alveolar macrophages by M.bovis strain AF2122/97 (MOI=10) infection at 2, 6, 24 and 48 hours post-infection is limited. The values of the M.bovis infected groups and non-infected group 6h, 24h and 48h were normalized to the mean of the non-infected group 2h (±SD). Student's *t*-test was used to evaluate the differences. The GEO file number is GSE62506 [66] (C) The activation bovine *NRAMP1* expression in monocyte-derived macrophages (MDM) by M.bovis strain M2137 (MOI=2) infection at 24h is limited. The values of the M.bovis infected group were normalized to the mean of the non-infected group (±SD). Student's *t*-test was used to evaluate the differences.The GEO file number is GSE45439 [67] (D) The expression of *Nramp1* in mouse bone marrow derived macrophages (BMDM) is significantly activated by Mtb strain H37Rv infection at 24h. The values of the H37Rv infected groups, non-infected group 24h were normalized to the mean of the non-infected group 4h (±SD). Student's *t*-test was used to evaluate the differences (*, $P < 0.05$). The GEO file number is GSE162620 [68]. The expression of *Nramp1* in alveolar macrophages of C57BL/6 mouse is significantly activated by Mtb strain H37Rv infection at 12h, 24h, 48h and 96h in vivo. The values of the H37Rv infected groups, non-infected group 24h, 48h, 96h and 120h were normalized to the mean of the non-infected group 12h (±SD). Student's *t*-test was used to evaluate the differences (**, $P < 0.01$). The GEO file number is GSE125287 [69]. (F) The

expression of *Nramp1* in RAW264.7 is significantly activated by Mtb strain H37Ra infection. The cells were infected with H37Ra (MOI=10) and then incubated for 4h, 12h, 24h, 36h and 48h. The expression of *Nramp1* was determined by qPCR. The values of the H37Ra infected groups, non-infected group 12h, 24h, 36h and 48h were normalized to the mean of the non-infected group 12h (±SD). Student's *t*-test was used to evaluate the differences (*, $P < 0.05$).

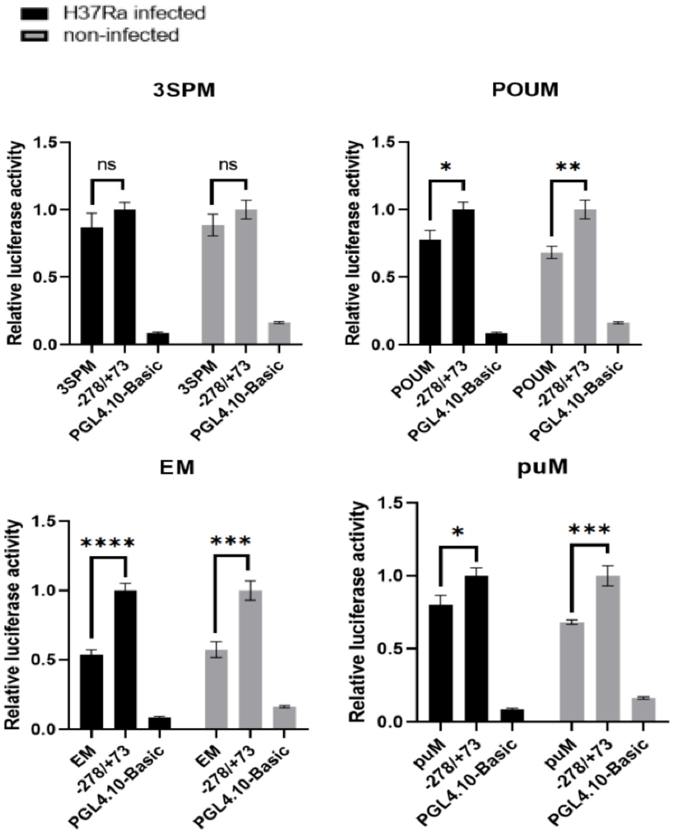

Supplementary Figure S2. Validation of the mutational effects at candidate editing sites. Relative transcriptional activities of mutated promoter 3SPM, POUM, EM and puM mutated promoters were determined by luciferase assays. pGL4.1 vectors that contained the different promoter fragments were co-transfected with pRL-TK in RAW264.7 respectively. The H37Ra infected groups were infected with H37Ra (MOI=10) 24h after transfection and then incubated for 24h. Values were normalized to the mean of the non-infected pGL4.1-T3(-278/+73) group (±SD). Student's *t*-test was used to evaluate the differences (*, $P < 0.05$;**, $P < 0.01$; ***, $P < 0.001$;****, $P < 0.0001$).

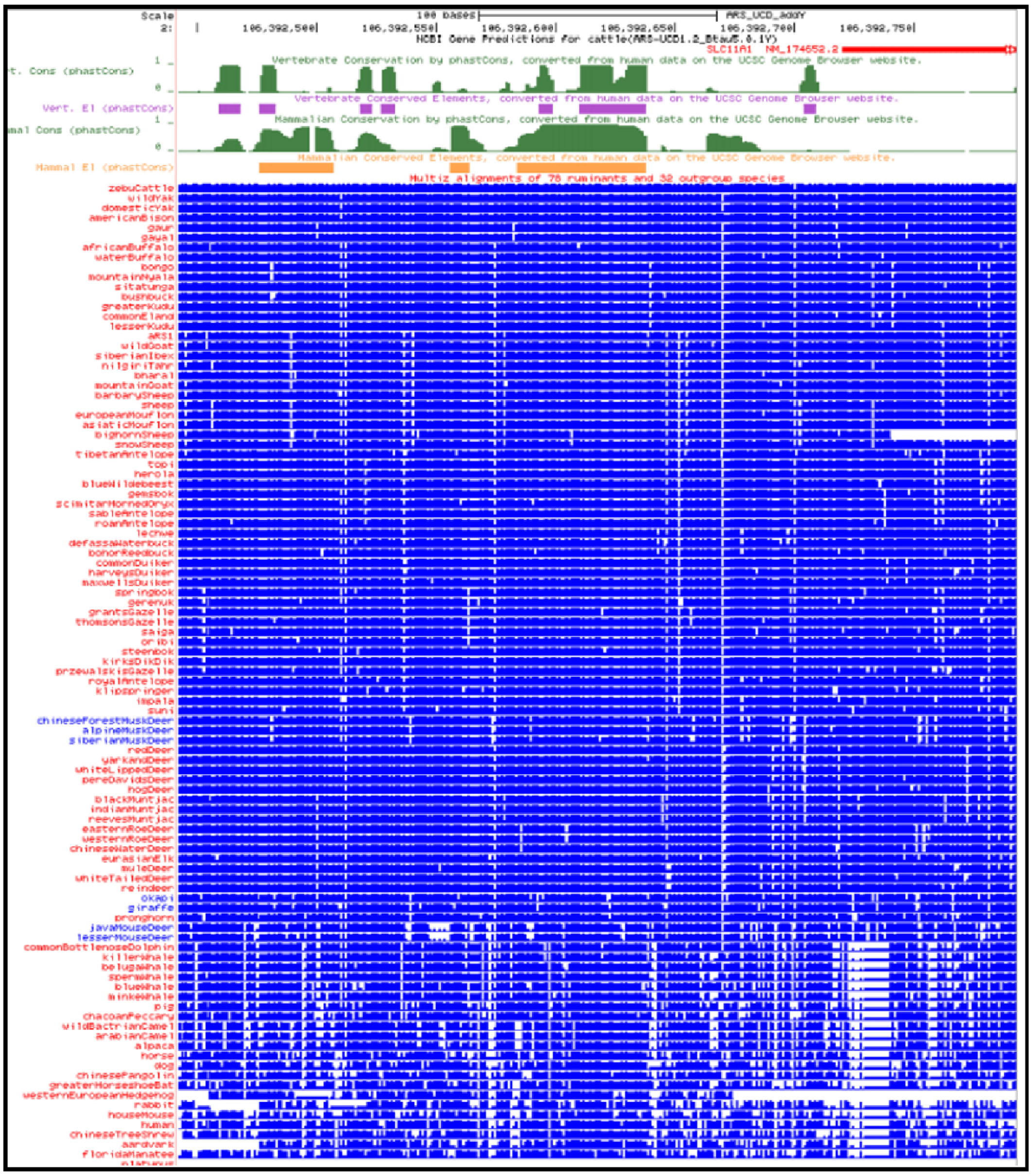

Supplementary Figure S3. Homology analysis of *NRAMP1* promoters between species. Species names are listed on the left. Blue indicates homologous sequences.

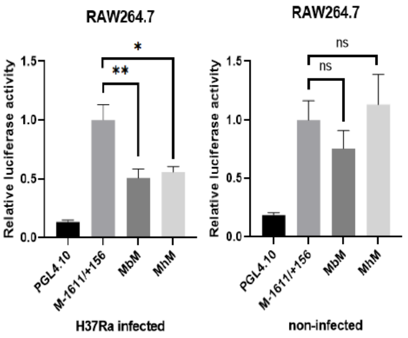

Supplementary Figure S4. Altering the 2SP site affinity for SP1/SP3 affects *NRAMP1* promoter activity. The substitution of 2SP site in the homologous region of the mouse *Nramp1* promoter significantly decreased the promoter activity after H37Ra infection. Relative transcriptional activities of the promoters were determined by luciferase assays. Values were normalized to the mean of the M-1611/+156 group (±SD). Student's *t*-test was used to evaluate the differences (*, $P < 0.05$; **, $P < 0.01$).

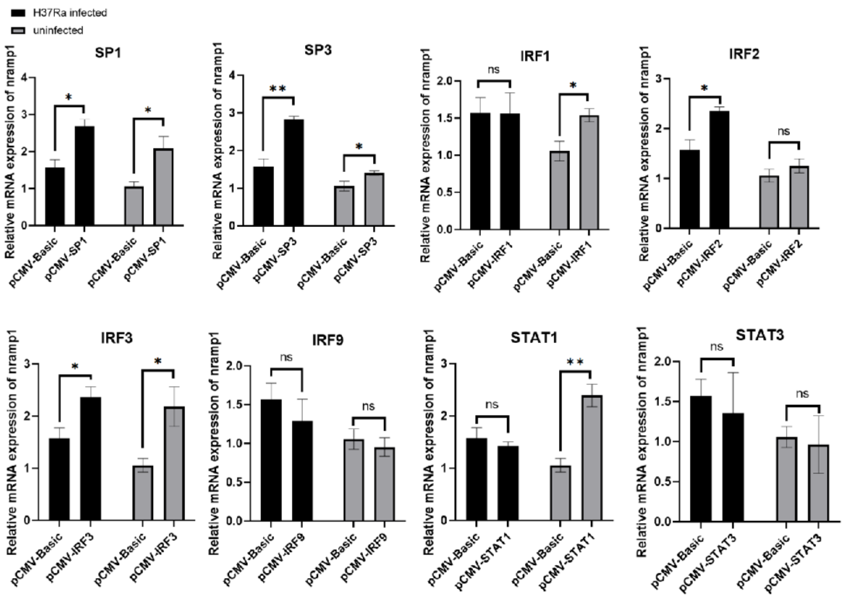

Supplementary Figure S5. Effects of different TFs overexpression in regulating *Nramp1* gene expression after H37Ra infected/non-infected. pCMV-SP1, pCMV-SP3, pCMV-IRF1, pCMV-IRF2, pCMV-IRF3, pCMV-IRF9, pCMV-STAT1, pCMV-STAT3 and pCMV-Basic (NC group) were transfected into RAW264.7 cells with or without H37Ra infection. The infected group cells were infected with H37Ra (MOI=10) at 24h after transfection. Expressions of *Nramp1* were detected by qPCR.

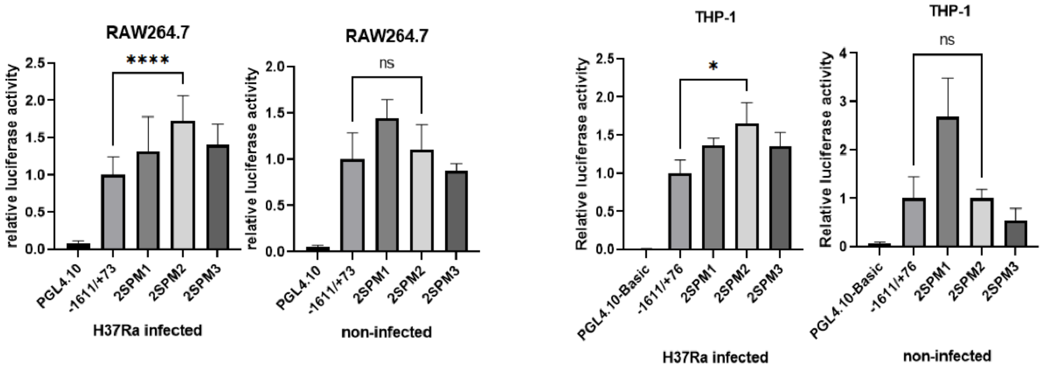

Supplementary Figure S6. The effects of different optimization mutations on the promoter activity after H37Ra infection or in uninfected conditions. RAW264.7 cells and THP-1 cells were used for verification respectively. Relative transcriptional activities of the promoters were determined by luciferase assays. Values were normalized to the mean of the -1611/+73 group (±SD). Student's *t*-test was used to evaluate the differences (*, $P < 0.05$; ****, $P < 0.001$).

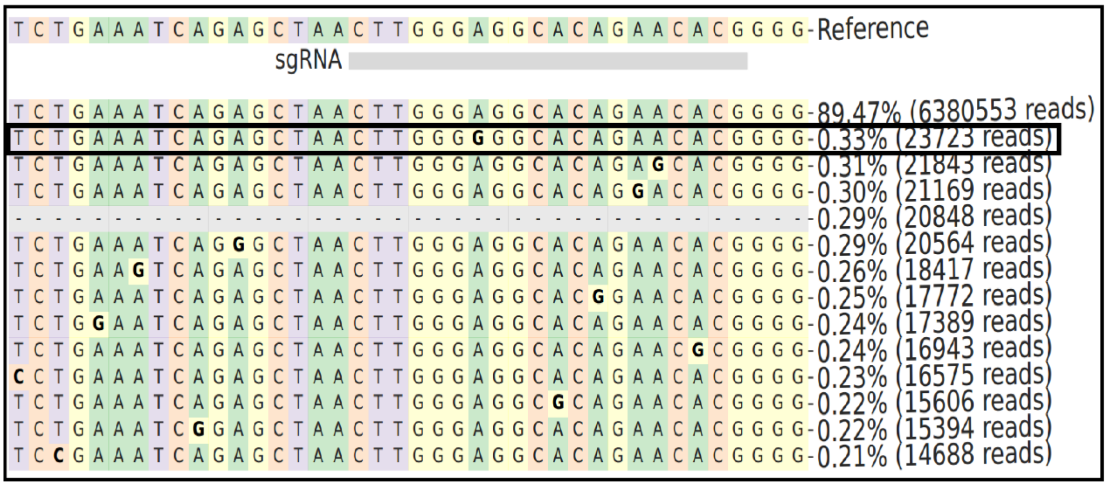

Supplementary Figure S7 frequency of precisely edited THP-1 cells. Sequences and proportions of precisely edited THP-1 cells are marked with black boxes.

Table S1 qPCR primers used in this study

name	Primer sequence （5' to 3', F: forward，R： reverse） and restriction site（underlined）
m-*gapdh*	F: AGGTCGGTGTGAACGGATTTG<br>R: TGTAGACCATGTAGTTGAGGTCA
m-*Nramp1*	F: TTACTCACTCGGACCAGCAC<br>R: GGGGGCTCTTGTCACTAATCA
m-*sp1*	F: GCCACCATGAGCGACCAAGA<br>R: AGTCTGAGAAAAGGCGGCAC
m-*sp3*	F: GTTTCCCGCACAGTCAATCA<br>R: CTTTTCGGGAGCGGTCATA
h-*Gapdh*	F: GAGTCAACGGATTTGGTCGT<br>R: GACAAGCTTCCCGTTCTCAG
h-*NRAMP1*	F: GCGTTCAACATCTGTGCCAA<br>R: CCTCAGGAAGCCCTCCATCA

Table S2 Mutated sequence of 7 candidate editing sites in bovine *NRAMP1* promoter

name	Site sequence（WT） and mutated sequence （MU）	Binding TFs
POUM	WT: 5'-AGACATGCATGCCCAAGTGGCAGGAA-3'	POU2F1
	MU: 5'-AGACA**ATTT**TGCCCAAGTGGCAGGAA-3'	
etsM	WT: 5'-AGACATGCATGCCCAAGTGGCAGGAAGTTG-3'	ETS
	MU: 5'-AGACATGCATGCCCAAGTGGCA**AAG**AGTTG-3'	
1SPM	WT: 5'-AGACATGCATGCCCAAGTGGCAGGAAGTTGTC-3'	SP1/SP3
	MU: 5'-AGACATGCATGCCCAA**ACAA**CAGGAAGTTGTC-3'	
2SPM	WT: 5'-TCAGAGCTAACTTGGGAGAGGTGCAGAACTCA-3'	SP1/SP3
	MU: 5'-TCAGAGCTAACTT**AAAAAAAT**TGCAGAACTCA-3'	
3SPM	WT: 5'-GGGCTCAGGCCAGGAGGGGAACAAATGTCC-3'	SP1/SP3
	MU: 5'-GGGCTCAGGCCA**TTAAAT**GAACAAATGTCC-3'	
EM	WT: 5'-AGACATGCATGCCCAAGTGGCAGGAA-3'	MYC
	MU: 5'-AGACATGCATGCC**ATAAAA**GCAGGAA-3'	
puM	WT: 5'-GAACTGGCCACTTCTGCCTTTGGAAA-3'	PU.1
	MU: 5'-GAACTGGCCAC**AAAA**GCCTTTGGAAA-3'	

The underlined bases are core putative TF-binding sites; the mutations are represented in bold.

Table S3 primers for the 7 potential TF binding site mutations in bovine *NRAMP1* promoter fragments and the homologous region of the 2SP site substitution/deletion in mouse *Nramp1* promoter fragments

name	Primer sequence （F: forward，R：reverse） and restriction site（underlined）	restriction enzyme
POUM	F: GGGGTACCAGACAATTTTGCCCAAGTGGCAGGAA	Kpn I
	R: CCGCTCGAGCATGAGGACCGCAGGCT	Xho I
EM	F: GGGGTACCAGACATGCATGCCATAAAAGCAGGAAGTTGTC	Kpn I
	R: CCGCTCGAGCATGAGGACCGCAGGCT	Xho I
1SPM	F: GGGGTACCAGACATGCATGCCCAAACAACAGGAAGTTGTC	Kpn I
	R: CCGCTCGAGCATGAGGACCGCAGGCT	Xho I
2SPM	F: GGGGTACCAGACATGCATGCCCAAGTGGCAGGAAGTTGTCCAAAAT CAGAGCTAACTTAAAAAAATTGCAGAACTCA	Kpn I
	R: CCGCTCGAGCATGAGGACCGCAGGCT	Xho I
3SPM	F1: GGGGTACCAAGTGGCAGGAAGTTGTC	Kpn I
	R1: AAGGACATTTGTTCATTTAATGGCCTGAGCCCCA	/
	F2: TGGGGCTCAGGCCATTAAATGAACAAATGTCCTT	/
	R2: CCGCTCGAGCATGAGGACCGCAGGCT	Xho I
etsM	F: GGGGTACCAGACATGCATGCCCAAGTGGCAAAGAGTTGTCCAAAAT	Kpn I
	R: CCGCTCGAGCATGAGGACCGCAGGCT	Xho I
puM	F1: GGGGTACCGGGAACAAATGTCCTTGCT	Kpn I
	R1: TTTCCAAAGGCTTTTGTGGCCAGTTC	/
	F2: GAACTGGCCACAAAAGCCTTTGGAAA	/
	R2: CCGCTCGAGCATGAGGACCGCAGGCT	Xho I
2SPM1	F1: GGGGTACCTCTGCCTTTGGAAAGTGTT	Kpn I
	R1: CACCCCCCCCAAGTTAGC	/
	F2: GCTAACTTGGGGGGGGTG	/
	R2: CCGCTCGAGCATGAGGACCGCAGGCT	Xho I
2SPM2	F1: GGGGTACCTGAGGATGGCTAAGAGGG	Kpn I
	R1: CACCTCTTTTAAGTTAGC	/
	F2: GCTAACTTAAAAGAGGTG	/
	R2: CCGCTCGAGCATGAGGACCGCAGGCT	Xho I
2SPM3	F1: GGGGTACCTGAGGATGGCTAAGAGGG	Kpn I
	R1: TGAGTTCTGCAAAGTTAGCTCTGATTTTG	/
	F2: AGCTAACTT~~GGGAGAGG~~TGCAGAACTCAGAGTGCCT	/
	R2: CCGCTCGAGCATGAGGACCGCAGGCT	Xho I
MbM	F1: GGGGTACCCACATCCTTACTTACCACATC	Kpn I
	R1: ATTCTTTGCCTCTCCCAAATTACCT	/
	F2: AGGTAATTTGGGAGAGGCAAAGAAT	/
	R2: CCGCTCGAGGAGGACGCAGGCGGCAGGATACT	Xho I
MhM	F1: GGGGTACCCACATCCTTACTTACCACATC	Kpn I
	R1: ATTCTTTGCCTCCCAAATTACCT	/
	F2: AGGTAATTTGGGAGGCAAAGAAT	/
	R2: CCGCTCGAGGAGGACGCAGGCGGCAGGATACT	Xho I

In overlap PCR, the two pairs of primers used are labeled as F1/R1 and F2/R2, respectively; The bases that have been replaced are highlighted in red, and the deletion line indicates that the sequence has been deleted.

Table S4 Optimized editing sequences of 2SP site in bovine *NRAMP1* promoter

name	the 2SP site sequence
WT	5’-GCTAACTTGGGAGAGGTG-3’
2SPM1	5’-GCTAACTTGGG**G**G**G**GGTG-3’
2SPM2	5’-GCTAACTT**AAA**AGAGGTG-3’
2SPM3	5’-GCTAACTT~~GGGAGAGG~~TG-3’

The mutations are represented in the uppercase; The strike-out line indicates the sequence has been deleted.
